# Coexistence of Heavy Metal Tolerance and Antibiotic Resistance in Thermophilic Bacteria Belonging to Genus *Geobacillus*

**DOI:** 10.3389/fmicb.2022.914037

**Published:** 2022-08-25

**Authors:** Ishfaq Nabi Najar, Sayak Das, Santosh Kumar, Prayatna Sharma, Krishnendu Mondal, Mingma Thundu Sherpa, Nagendra Thakur

**Affiliations:** ^1^Department of Microbiology, Sikkim University, Gangtok, India; ^2^Department of Microbiology, Vidyasagar University, Midnapore, India

**Keywords:** thermophilic microbes, antibiotics resistance, heavy metal tolerance, *Geobacillus*, Sikkim Himalaya

## Abstract

Hot springs are thought to be potential repositories for opportunistic infections, such as antibiotic-resistant strains. However, there is a scarcity of information on the mechanisms of antibiotic resistance gene (ARG) uptake, occurrence, and expression in thermophilic bacteria. Furthermore, because the genesis and proliferation of ARGs in environmental microorganisms are unknown, the research on antibiotic resistance profiles and probable mechanisms in thermophilic bacteria will become increasingly important. The goals of this study are to explore bacterial diversity, antibiotic and heavy metal resistance, and the prevalence and presence of ARG and metal resistance gene (MRG) in *Geobacillus* species. The 16S rRNA sequencing was used to determine the culturable bacterium diversity of 124 isolates. Standard Kirby Bauer Disc Diffusion and tube dilution procedures were used to determine antibiotic sensitivity and minimum inhibitory concentration (MIC). The tube dilution method was also used to check metal tolerance. To detect ARG and heavy MRG (HMRG), whole genome sequencing studies of the type species of the genus *Geobacillus* and five randomly selected *Geobacillus* species were performed. Graph Pad Prism and XLSTAT were used to perform statistical analyses such as ANOVA, EC50 analysis, and principal component analysis (PCA). The phylum Firmicutes and the genus *Geobacillus* dominated the culture-dependent bacterial diversity. Surprisingly, all thermophilic isolates, i.e., *Geobacillus* species, were sensitive to at least 10 different antibiotics, as evidenced by the lack of ARGs in whole genome sequencing analysis of numerous *Geobacillus* species. However, some of these isolates were resistant to at least five different heavy metals, and whole genome sequencing revealed the presence of MRGs in these thermophilic bacteria. The thermophilic genus *Geobacillus* is generally antibiotic sensitive, according to this study. In contrast, heavy metal is tolerated by them. As a result, it is possible that ARGs and MRGs do not coexist in these bacteria living in hot springs.

## Introduction

Antibiotics are a type of chemotherapeutic medication that suppresses or kills microorganisms (pathogens) by interacting with their targets in a specific way (Dafale et al., 2016). Antibiotics are extremely important in the fight against infectious diseases produced by germs. In contrast, antibiotic resistance in microorganisms is a serious problem and a hazard in today's society, resulting in inoperable infections, mortality, and rising healthcare expenditures (Miller et al., 2016). The essential determining pressure (regardless of the existence of antibacterial agents) that drives cumulative rates of resistance is eventually found in the abuse and misuse of antibacterial agents, whether in inappropriate prescribing, extensive agricultural use, availability of few antibiotics or regulatory barriers, or patient non-compliance with the prescribed antibiotic course (Roca et al., 2015; Ventola, 2015). Bacteria may accumulate numerous resistance characteristics over time, making them resistant to several antibiotic classes (Levy, 1992).

Antibiotic resistance has been widely explored in pathogenic and non-pathogenic bacteria belonging to the mesophiles. In contrast, antibiotic resistance in thermophilic bacteria has received far less attention from researchers (Najar et al., 2020a). As a result, the following question may arise: what if thermophilic bacteria develop resistance and become pathogenic to humans? Thermophilic bacteria are being actively used in biotechnological and industrial domains, making it a rising concern. The most concerning aspect is that we have less knowledge of the mechanisms governing the acceptance, occurrence, and expression of numerous genes associated with antibiotic resistance in thermophilic bacteria (Najar et al., 2020b).

Aside from antibiotic resistance, heavy metal resistance is a significant issue because of its harmful effect and buildup throughout the food chain, resulting in substantial ecological and health issues (Iyer et al., 2005). The coexistence of heavy metals and antibiotics, which is thought to occur in many natural ecosystem matrices, has posed a greater concern. Combined contaminations of heavy metals and antibiotics in some natural habitats with microbial populations contribute to the occurrence and spread of microbial antibiotic resistance and multidrug resistance (Peltier et al., 2010). As a result, it is critical to investigate antibiotic resistance and heavy metal resistance in thermophilic bacteria and their co-occurrence. There are several factors to consider while selecting thermophiles for this type of research.

Thermophiles are most prevalent in hot springs, geysers, and fumaroles. Hot springs are frequently associated with techniques, such as balneotherapy or hydrotherapy, and locals regard them as sacred entities with varied therapeutic and healing characteristics (Nasermoaddeli and Kagamimori, 2005), in addition to their aesthetic appeal. Aside from this, hot springs and thermal baths have been demonstrated to harbor opportunistic infections (Singh et al., 2013). In contrast, infections acquired from hot springs are rare. Rabkin et al. (1985) discovered that thermophilic bacteria with an optimal development temperature of 50°C can cause diseases in humans, such as meningitis, endocarditis, and septicemia (Rabkin et al., 1985). Furthermore, numerous antibiotics, including erythromycin, tetracycline, sulfamethoxazole, tobramycin, and netilmicin, were found to be resistant to these thermophilic isolated bacteria (Rabkin et al., 1985). Another study found that thermophilic bacteria, such as *Arthrobacter* sp. and *Hafnia* sp., which were identified as opportunistic food-borne pathogens, were antibiotic-resistant (Jardine et al., 2017). As a result, hot springs could be potential repositories for new opportunistic diseases, such as antibiotic-resistant strains. As a result, it is critical to look at the antibiotic resistance profiles of these thermophilic bacteria.

- The second goal of this research is to determine the evolutionary relationship between antibiotic resistance genes (ARGs) and thermophiles. The evolution and spread of antibiotic resistance in opportunistic organisms is a major issue that has sparked debate. Environmental species are the store of these resistance genes, according to different research (Dcosta et al., 2011; Bhullar et al., 2012). In contrast, other studies show that anthropogenic use played a role in the development of these genes (Knapp et al., 2010; Thaller et al., 2010). As a result, the breadth of the research to determine the profile of antibiotic resistance and possible mechanisms will be explored. Because hot springs are regarded as isolated habitats with primitive prokaryotic communities, such as bacteria and archaea, it will be fascinating to look into the resistance profile of thermophilic bacteria isolated from them.
- The third argument in favor of examining the antibiotic resistance profile of hot springs is that several studies on sewage sludge and dairy manure have revealed that raising temperature reduces the number of resistance genes (Sun et al., 2016; Jang et al., 2018). However, recent research has found that as the temperature rises, so does antibiotic resistance (MacFadden et al., 2017). Because the temperatures in Sikkim's hot springs range from 45 to 75°C, it is fascinating to compare the prevalence of antibiotic resistance in different hot springs with varying temperatures and thereby correlate the effect of temperature on antibiotic resistance. Functional metagenomics could add to this paradigm and bring up new research avenues. For example, if some bacterial communities in the same hot spring retain resistance genes at higher temperatures than other bacterial communities, this raises two research questions, namely, (i) how these bacteria could retain resistance genes, or are there any novel mechanisms of resistance involved, and (ii) if susceptible bacteria are abundant, are they devoid of competition or are they able to produce any unknown or novel protecting mechanisms.
- Finally, the creation of data may be the most important aspect of this study. Because thermophiles are less understood in terms of antibiotic and heavy metal resistance, there is a scarcity of data on their resistance profile. Using metagenomic techniques, we previously investigated the frequency of antibiotic resistance in hot springs (Najar et al., 2020a). Antibiotic resistance was not found in the hot springs, according to the study. However, no culture-dependent data on antibiotic susceptibility test (AST) or minimum inhibitory concentration (MIC) ranges of antibiotics ever used in thermophiles are available. As a result, our research could aid in the creation of a dataset on the resistance profile of thermophilic bacteria, particularly those of the *Geobacillus* genus. We opted to check the antibiotic profile of the genus *Geobacillus* using culture-dependent methods and evaluating entire genome sequences of type *Geobacillus* species because *Geobacillus* is the most common resident of hot springs.

In the context of Sikkim, the study described earlier was possible due to the state's abundance of natural hot springs of varying temperatures. These hot springs are similarly spread out across a large geographical area. In addition, a large number of people visit Sikkim's hot springs for a variety of reasons, including drinking and cooking (Das et al., 2012; Najar et al., 2018). To examine antibiotic resistance in bacteria living in these hot springs, it was necessary to consider possible side effects and the emergence of ARGs.

This study used culture-dependent and culture-independent methodologies to examine the prevalence and abundance of bacterial variety, antibiotic and metal resistance, and the co-occurrence of antibiotic and metal resistance.

## Materials and Methods

### Sample Collection

This article delves into water samples from Sikkim's four hot springs, namely, Polok, Borong, Reshi, and Yumthang. Temperature, pH, dissolved oxygen (DO), total dissolved solids (TDS), and conductivity were measured *in situ* at the sampling location using the Multi Water Quality Checker U-50 Series (Horiba, Japan). The water samples were collected aseptically in triplicates in sterile thermal flasks (Mega Slim, USA) for microbiological testing (culture dependent). The samples were then promptly transported to the laboratory, processed, and stored at 4°C.

### Culture-Dependent Technique

#### Isolation and Basic Morphological Characterization of Bacterial Strains

The thermophilic bacteria were isolated using the conventional spread plate and streak plate procedures using various media, such as Thermus media (peptone 85 g L^−1^, yeast extract 4 g L^−1^, NaCl 2 g L^−1^, and agar 25 g L^−1^) and R2A media (casein acid hydrolysate 0.5 g L^−1^, yeast extract 0.5 g L^−1^, proteose peptone 0.5 g L^−1^, dextrose 0.5 g L^−1^, starch soluble 0.5 g L^−1^, dipotassium phosphate 0.3 g L^−1^, magnesium sulfate 0.024 g L^−1^, sodium pyruvate 0.3 g L^−1^, and agar 25 g L^−1^) (Brumm et al., 2016; Mohammad et al., 2017) (Supplementary Table 1). All of the bacteria were isolated at 60°C for 24–72 h. Following incubation, various colonies were chosen based on their morphological traits and purified using the subculturing process. Bacterial strains were isolated and purified and kept at −80°C in a 50% glycerol stock. Gram staining and spore staining were performed using Gram stain kit (Himedia, Mumbai India) K001-1KT, and Schaeffer and Fulton's spore stain kit, respectively. Bacterial smears were made in clean grease-free slides. It was air-dried and then heat fixed. Slides were then placed over a hot water bath for 10 min (with the bacterial film on the upper side). When large droplets condense on the lower side of the slide, the slide was flooded with Schaeffer and Fulton's spore stain A and then steamed for 3–6 min. Then, the slide was rinsed under running tap water and counterstained with Schaeffer and Fulton's spore stain B for 30 s. The slides were then washed with water. After drying, the slides were observed under an oil emersion lens 100× light microscope. The protocol was given by the kit manufacturer.

### DNA Extraction, Purification, and 16S rRNA Sequencing

Total genomic DNA was extracted using the Qiagen QIAamp DNA Mini Kit 50 according to the manufacturer's instructions. The 16S rRNA gene was amplified using two universal primers, namely, 27F (5′-AGAGTTTGATCMTGGCTCAG-3′) and 1406R (5′-GACGGGCGGTGTGTRCA-3′) (Baker and Cowan, 2004; Devereux and Wilkinson, 2004). The reaction mixture and PCR reaction parameters were set up according to earlier research (Najar et al., 2018). The Applied Biosystems' BigDyeTMTerminator version 3.1 cycle sequencing kit was used to sequence the samples on an AB3500 Genetic Analyzer. The Clustal W software was used to match the 16S rRNA sequences with representative sequences from similar taxa (Thompson et al., 1994). Using the MEGA 11 and FigTree software, a phylogenetic tree was created using the maximum likelihood method (Tamura et al., 2021) and Jukes-Cantor evolutionary distance measurement (Erickson, 2010).

### Antibiotic Sensitivity of Isolates

The antibiotic sensitivity of the bacterial isolates was determined using the Kirby Bauer Disc Diffusion technique. In Mueller Hinton agar (MHA) plates, 0.1 ml of the culture broth was swabbed with sterile cotton, and the sterile antibiotic disc was placed aseptically on the swabbed plate. The negative control was created by swabbing the swabbed plate with sterile 0.4 m membrane filter paper discs soaked in sterile autoclaved water. The antibiotic sensitivity of a single isolate was tested using 10 different antibiotics, namely, ampicillin (10 μg), penicillin (10 U), methicillin (10 μg), amoxicillin (10 μg), erythromycin (15 μg), chloramphenicol (30 μg), gentamycin (10 μg), clindamycin (2 μg), norfloxacin (10 μg), and norfloxacin (10 μg). The diameter of the zone of inhibition on the MHA plates was measured after 48 h of incubation at 60°C. The susceptibility tests were performed according to CLSI guidelines (Nguyen et al., 2016).

### Minimum Inhibitory Concentration (MIC)

The minimum inhibitory concentration of various antibiotics used was carried out by standard methods (Mazzola et al., 2009). The MIC of penicillin, vancomycin, erythromycin, chloramphenicol, and methicillin was checked. The antibiotic stock solution was prepared according to the following formula:


(1) $$\begin{array}{l} \;\text{W}\;=\;1,000/\left(\text{P}\;\times \;\text{V}\;\times \;\text{C}\right), \end{array}$$

where W is the weight of antibiotic to be dissolved in V, V is the volume required (in ml), P is the potency (already given on antibiotic pack), and C is the final concentration of the solution in multiples of 1,000. The stock solutions were freshly prepared and kept at 4°C for later use. Later, various dilutions from the stock antibiotic solution, such as 0.25, 0.5, 1, 2, 4, 8, and 16 mg L^−1^, were prepared.

No internationally accepted criteria for susceptibility testing or breakpoints for susceptible or resistant isolates are available for thermophilic bacteria. However, the two thermophilic *Campylobacter* species were investigated and their breakpoints have been established (Guévremont et al., 2006). Briefly, the breakpoint values of the MIC for resistance were as follows: for ciprofloxacin, clindamycin, and enrofloxacin, ≥4 μg ml^−1^; for erythromycin, ≥8 μg ml^−1^; for gentamicin and tetracycline, ≥16 μg ml^−1^; for ampicillin and chloramphenicol, ≥32 μg ml^−1^; for streptomycin, ≥ 64 μg ml^−1^; and for sulfamethoxazole, ≥512 μg ml^−1^ (Guévremont et al., 2006).

### Screening and Assessment of Metal Toxicity

Only two hot springs, Reshi and Yumthang, were chosen as representatives from two areas in Sikkim for investigating this metal toxicity since the heights and geographical or climatic circumstances of these two hot springs are so different. A total of 27 bacterial isolates from the Reshi and Yumthang hot springs were screened and analyzed for metal toxicity (based on morphological and biochemical characterization). CuSO_4_ (0.5–15 mM), MnSO_4_ (0.05–3 mM), ZnCl_2_ (1–10 mM), HgCl_2_ (0.005–1 mM), and CoCl_2_ (0.05–2 mM) were used to make heavy metal solutions from their chloride and sulfate salts. Stock solutions were made in distilled water and gently acidified with HNO_3_ before being autoclaved and stored at 4°C for no more than 1 month. The MIC values were checked using the tube dilution method (Hassen et al., 1998). After incubation at 60°C for 24 h, tubes were read at 600 nm in a UV-Vis spectrophotometer (Perkin Elmer LAMBDA 40) (Kim et al., 2007).

### Statistical Analysis

The statistical analysis Graph Pad Prism software was used to analyze the variance (ANOVA). The effective concentration (EC) values (a statistically obtained estimate of a chemical concentration that results in a 50% reduction in growth within a given time) were calculated. Three replicate samples were used to obtain the data. XLSTAT 2014.03 was used to perform principal component analysis (PCA).

### PCR-Based Detection of ARGs

The PCR-based detection of ARGs was carried out for ampicillin, penicillin, and methicillin antibiotics. In the case of penicillin, *pbp*1A and *pbp*2A genes were amplified using *pbp*1F-(5′-CCAGCAACAGGTGAGAGTC-3′) and *pbp*1R-(5′-GTAAACACAAGCCAAGAC AC-3′) primers (Sanbongi et al., 2004). For ampicillin, the *amp*C gene was amplified using *amp*C F-(5′-TGAGTTAGGTTCGGTCAGCA-3′) and *amp*C R-(5′-AGTATTTTGTTGCGGGATCG-3′) primers (Fernando et al., 2016), and for methicillin, *mec*A1 and *mec*A2 genes were amplified using *mec*A1-(5′-AAAATCGATGGTAAAGGTTGGC-3′) and *mec*A2-(5′-AGTTCTGCAGTA CCGGATTTGC-3′) primers (Cuteri et al., 2003) (Tables 1A,B).

**Table 1A:** Principal Component Analysis (correlation between heavy metals and isolates): Eigenvalues.

	**F1**	**F2**	**F3**	**F4**	**F5**
Eigen value	2.0710	1.1150	0.8174	0.6670	0.3296
Variability (%)	41.4206	22.3003	16.3476	13.3404	6.5911
Cumulative (%)	41.4206	63.7209	80.0685	93.4089	100.0000

**Table 1B:** Bartlett's sphericity test.

**Chi-square (Observed value)**	**23.3116**
Chi-square (Critical value)	18.3070
DF	10
*p*-value	**0.0097**
alpha	0.05

*Bold values indicate the maximum EC50 value obtained for a strain with respect to the heavy metal concentration*.

### ARG and MRG Analysis by Whole Genome Sequencing

Five culturable isolates were chosen at random for the whole genome sequencing study to examine ARG and heavy metal resistance gene (HMRG). The NCBI GenBank under accession numbers of various isolates used in this study has been mentioned such as the accession number of *Geobacillus yumthangensis* AYN2 is NWUZ00000000, *Parageobacillus* sp. strain SY1 is VRMH00000000, *Geobacillus* sp. AYS3 is VRMI00000000, and *Geobacillus* sp. LYN3 is QCWL00000000. The Illumina HiSeq 4000 sequencing technology with a paired-end sequencing module was used to sequence the whole genome. Annotation and functional characterization of the draft genomes were completed. Rapid Annotations using Subsystems Technology (version 2.0) were the annotation tool used (Aziz et al., 2008).

### ARGs and MRGs of *Geobacillus* Species

These type strains and our 5 isolates were also analyzed using four pipelines, namely, CARD (https://card.mcmaster.ca/home) (Alcock et al., 2020), Galaxy with two applications, Galaxy ARGA and Galaxy ABRicate (Afgan et al., 2018), and ARG-ANNOT (http://backup.mediterranee-infection.com/article.php?laref=282&titre=arg-annot) (Gupta et al., 2014). BacMetScan version 1.0 http://bacmet.biomedicine.gu.se/ was used to identify possible MRGs. The BacMet AntiBacterial Biocide and Metal Resistance Gene Database have a script (Pal et al., 2014) available. The manually curated database of genes with experimentally confirmed resistance function was used in BacMet-Scan as a reference. The predicted resistance genes have been collected using similarity searches in the NCBI non-redundant database. The NCBI protein database was taken as a reference database.

## Results

### Sampling and Analysis of Physical Parameters

Sikkim's hot springs are found in the Himalayan Geothermal Belt (HGB). Reshi, Polok, and Borong hot springs are located on the banks of the river Rangit in the South Sikkim area, while Yumthang hot spring is located in Yumthang valley alongside the river Lachung in North Sikkim. Physical parameters were evaluated prior to sampling, and it was discovered that the Polok hot spring was hotter than the rest of the hot springs, with a temperature of 75–77°C, and that the pH of the Borong hot spring was somewhat alkaline, with a pH of 8. Table 2 shows that the temperature of Reshi was 47.4°C and that of Yumthang hot spring was 41°C.

**Table 2:** Physical Parameters of Polok, Borong, Reshi and Yumthang Hot Springs.

**Hot Spring**	**Temperature** **(in **°**C)**	**pH**	**Conductivity** **(mS cm^**−1**^)**	**D.O.** **(mg L^**−1**^)**	**D.O.** **(%)**	**TDS** **(g L^**−1**^)**
Polok	76.3	7.52	0.756	5.56	92	0.483
Borong	52.3	5.32	0.205	6.56	98.3	0.133
Reshi	47.4	6.57	0.935	7.07	104.09	0.598
Yumthang	41	7.5	0.234	7.5	116.5	0.15

### Culture-Dependent Diversity

#### Identification of Bacteria and Phylogenetic Analysis

Around 200 isolates were isolated from the four hot springs, with 124 being selected based on morphological features. The cell shape of bacteria suggested that the majority of the isolates were Gram-positive rods and spore formers; however, some of them had Gram variable reactions (Supplementary Tables 2a,b,c).

**Figure 1:**
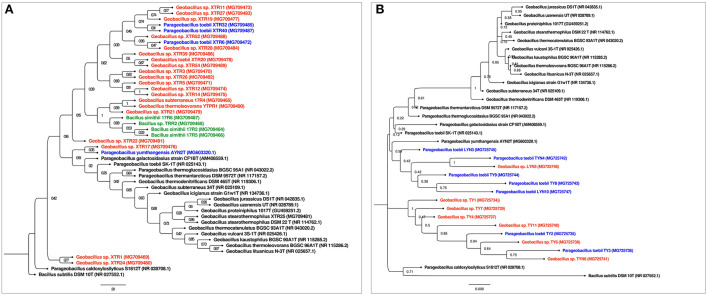
**(A)** Phylogenetic tree of Reshi isolates: The evolutionary history was inferred using the Maximum Likelihood method. The bootstrap consensus tree inferred from 500 replicates is taken to represent the evolutionary history of the taxa analyzed. The evolutionary distances were computed using the Jukes-Cantor method and are in the units of the number of base substitutions per site. Evolutionary analyses were conducted in MEGA11. *Bacillus subtilis* DSM 10T (NR 027552.1) was taken as an outgroup. *Geobacillus* isolates were colored red and *Parageobacillus* isolates were colored as blue. **(B)** Phylogenetic tree of Yumthang isolates: The evolutionary history was inferred using the Maximum Likelihood method. The bootstrap consensus tree inferred from 500 replicates is taken to represent the evolutionary history of the taxa analyzed. The evolutionary distances were computed using the Jukes-Cantor method and are in the units of the number of base substitutions per site. Evolutionary analyses were conducted in MEGA11. *Bacillus subtilis* DSM 10T (NR 027552.1) was taken as an outgroup. *Geobacillus* isolates were colored red and *Parageobacillus* isolates were colored as blue.

The 16S rRNA partial sequencing showed the unusual dominance of phylum Firmicutes. The major genus found in our study was *Geobacillus* with a few representatives from other genera, such as *Anoxybacillus* and *Bacillus*. Identified isolates of *Geobacillus* were *Geobacillus stearothermophilus* XTR25, *Geobacillus kaustophilus* YTPR1, *Geobacillus subterraneus* 17R4, *Geobacillus lituanicus* TP11, various strains of *Geobacillus toebii* such as *G. toebii* TYN4, LYN10, LYN3, TY3, TY1, TP3, TP5, etc., *Parageobacillus toebii* 10PHP2, *G. kaustophillus* YTPB1, *Geobacillus* sp. BPP2, and *Geobacillus* sp. TB7. The representative isolates of the genus *Anoxybacillus* were *Anoxybacillus gonensis* TP9 and *Anoxybacillus caldiproteolyticus* TRB1. The representative isolates of the genus *Bacillus* were *Bacillus simithi* 17R6 and *Bacillus* sp. 17R5. In addition, some uncultured bacteria were reported such as *Bacillus smithii* 17R2 and *Bacillus* sp. TRR2 (Figures 1A,B). The alignment and similarity search of 16S rRNA partial sequencing with the nr/nt database of NCBI have shown that many of the isolates have a distinct Average Nucleotide Identity (ANI) < 95%.

### Antibiotic Susceptibility Test

The susceptibility of 10 antibiotics from seven distinct classes was tested using the Kirby Bauer disc diffusion method, -lactams, aminoglycosides, macrolides, quinolones, glycopeptides, lincosamides, and chloramphenicol are among the several classes. As indicated in Supplementary Tables 3a,b, we discovered that all of the isolates were sensitive to the various types of antibiotics utilized. According to CLSI, the zone of inhibition was also compared to Gram-positive bacteria *Staphylococcus aureus*. We also tested two common *Geobacillus* species, *G. stearothermophilus* (MTCC37) and *Geobacillus thermoleovorans* (MTCC4219), and found that they were susceptible to the same 10 antibiotics as the other *Geobacillus* species (Supplementary Table 3c).

### Minimum Inhibitory Concentration (MIC)

**Figure 2:**
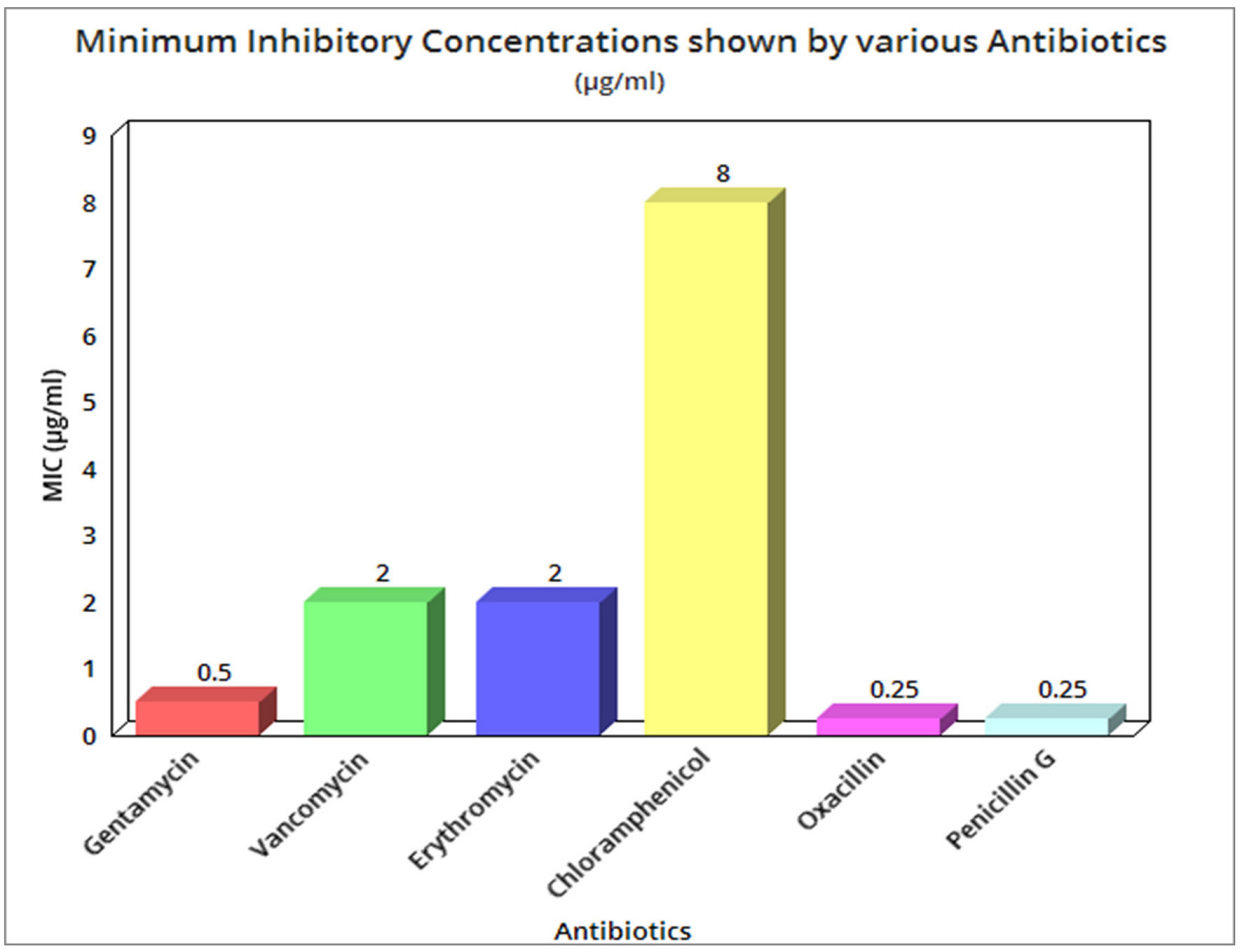
Minimum inhibitory concentration shown by various antibiotics in μg/ml.

The MIC values of bacterium isolates against penicillin, vancomycin, erythromycin, chloramphenicol, gentamycin, and oxacillin have been determined to be quite low. The MIC values for gentamycin, vancomycin, erythromycin, and chloramphenicol were, respectively, 0.5, 2, 2, and 8 g mL^−1^. In the case of penicillin G and oxacillin, the lowest MIC of 0.25 g ml^−1^ was found, as indicated in Supplementary Tables 4a,b,c,d, and Figure 2. For thermophilic bacteria, there are no widely established standards for susceptibility testing or breakpoints. The real sensitive character of our isolates was revealed by comparing MIC values with the only known report (Guévremont et al., 2006).

### PCR-Based Detection of ARGs

Our bacterial isolates' antibiotic susceptibility could be attributable to the lack of ARGs or the inhibition of ARG production. As a result, we first examined the presence of some prevalent ARGs in our isolates from the -lactam class. Penicillin-binding proteins PBP1A and PBP2A genes, ampicillin-resistant ampC genes, and methicillin-resistant mecA1 and mecA2 genes were all targeted. However, no PCR products were identified in the agarose gel electrophoresis, indicating that such genes were not present in cultured isolates (Supplementary Figure 1). ARGs are typically found on bacteria's plasmids. As a result, plasmids were isolated from 50 different bacterial isolates. As shown in Supplementary Figure 1, no plasmid DNA was found by agarose gel electrophoresis against the positive control, indicating that none of the isolates had plasmids.

### Screening of Metal Toxicity

**Figure 3:**
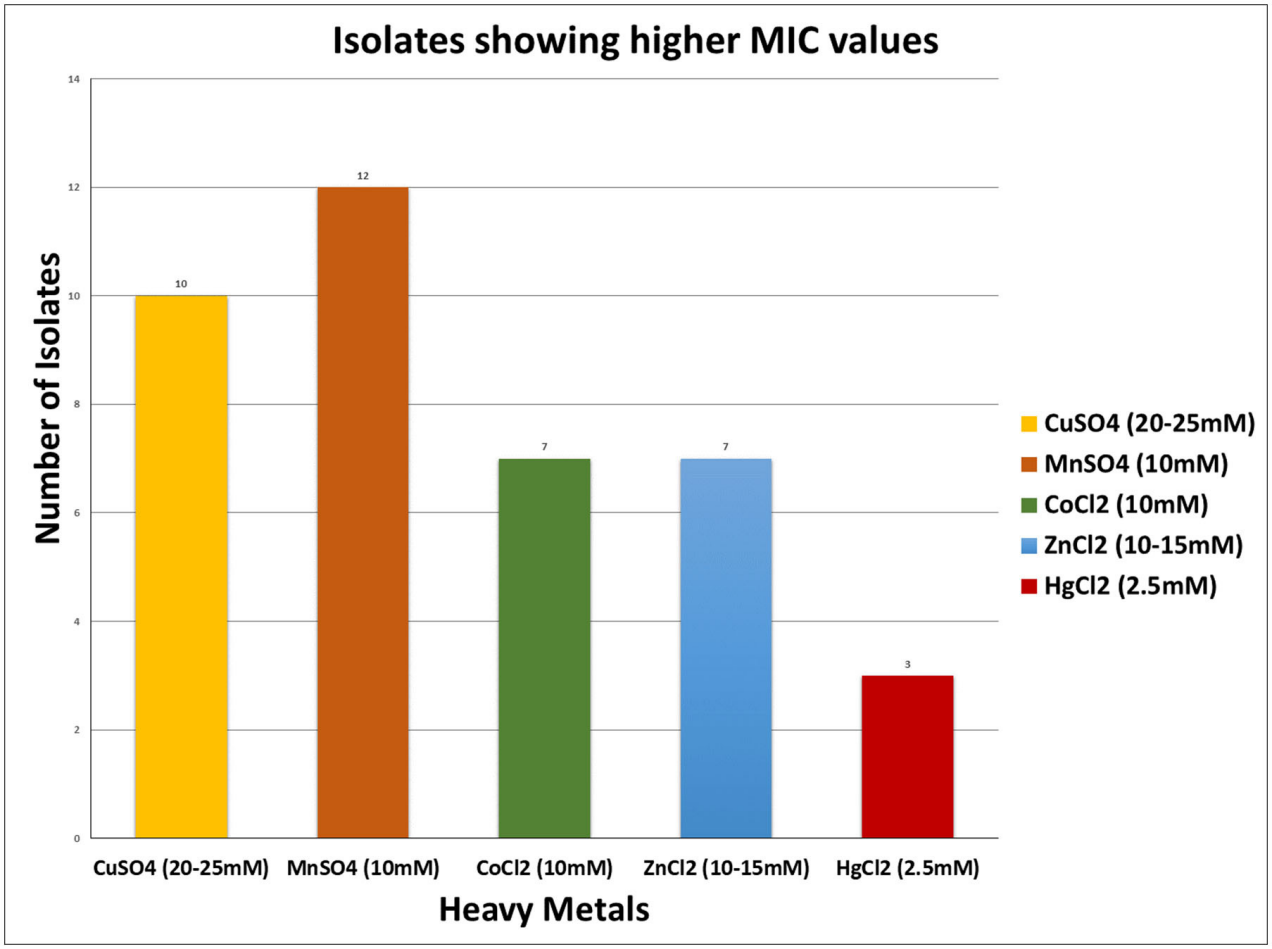
Minimum inhibitory concentration showing by isolates toward various heavy metals.

For almost all of the heavy metals tested, the data revealed considerable tolerance of MIC values. The MIC values for Cu, Mn, Co, Zn, and Hg were around 25, 15, 10, 15, and 2.5 mM, respectively. Hg had the lowest MIC value of 0.25 mM, followed by cobalt, as indicated in Table 3, Figure 3. The isolates with higher MIC values against a certain concentration of heavy metals also have EC50 values that match (Supplementary Table 5).

**Table 3:** Minimum Inhibitory Concentration of heavy metals (in liquid media).

**Minimum Inhibitory Concentration (in liquid media)**							
**Isolates**	**CuSO**_**4**_	**MnSO**_**4**_	**CoCl**_**2**_	**ZnCl**_**2**_	**HgCl**_**2**_	***R* ^2^**	***P*** **-value**
SY1	1.5	4	2	1.5	0.1	0.6	<0.0001
SY3	1.5	4	5	1.5	0.1		
SY4	1	4	2	1.5	0.1		
SY5	1.5	4	5	2	0.1		
SY6	1.5	10	5	2.5	0.1		
SY8	1.5	4	2	2.5	0.1		
SY12	1.5	4	2	2.5	0.1		
SY14	1.5	4	2	2.5	0.2		
SY15	1.5	4	2	2.5	0.2		
SY17	1.5	4	2	15	0.1		
AYS1	5	10	2	15	0.2		
AYS2	1.5	10	2	5.5	0.1		
AYS3	1.5	10	2	5.5	0.1		
AYS4	5	3	2	5.5	0.2		
AYS6	1.5	3	2	5.5	0.1		
AYS7	1.5	3	5	15	0.2		
AYS8	1.5	3	2	5.5	0.2		
AYS10	1.5	3	5	5.5	0.2		
AYS11	1.5	3	2	5.5	0.1		
AYS13	1.5	3	2	5.5	0.1		
XTR1	20	10	10	2.5	0.5		
XTR9	25	10	5	2.5	0.5		
17R2	25	10	5	2.5	0.5		
TRR2	20	15	10	2.5	0.5		
XTR15	25	10	5	2.5	0.5		
XTR10	20	10	10	10	0.5		
XTR25	25	15	10	10	0.5		
YTPR1	20	10	10	10	2.5		
17R4	20	10	10	5	2.5		
17R5	25	10	10	10	2.5		

### Principal Component Analysis (PCA)

**Figure 4:**
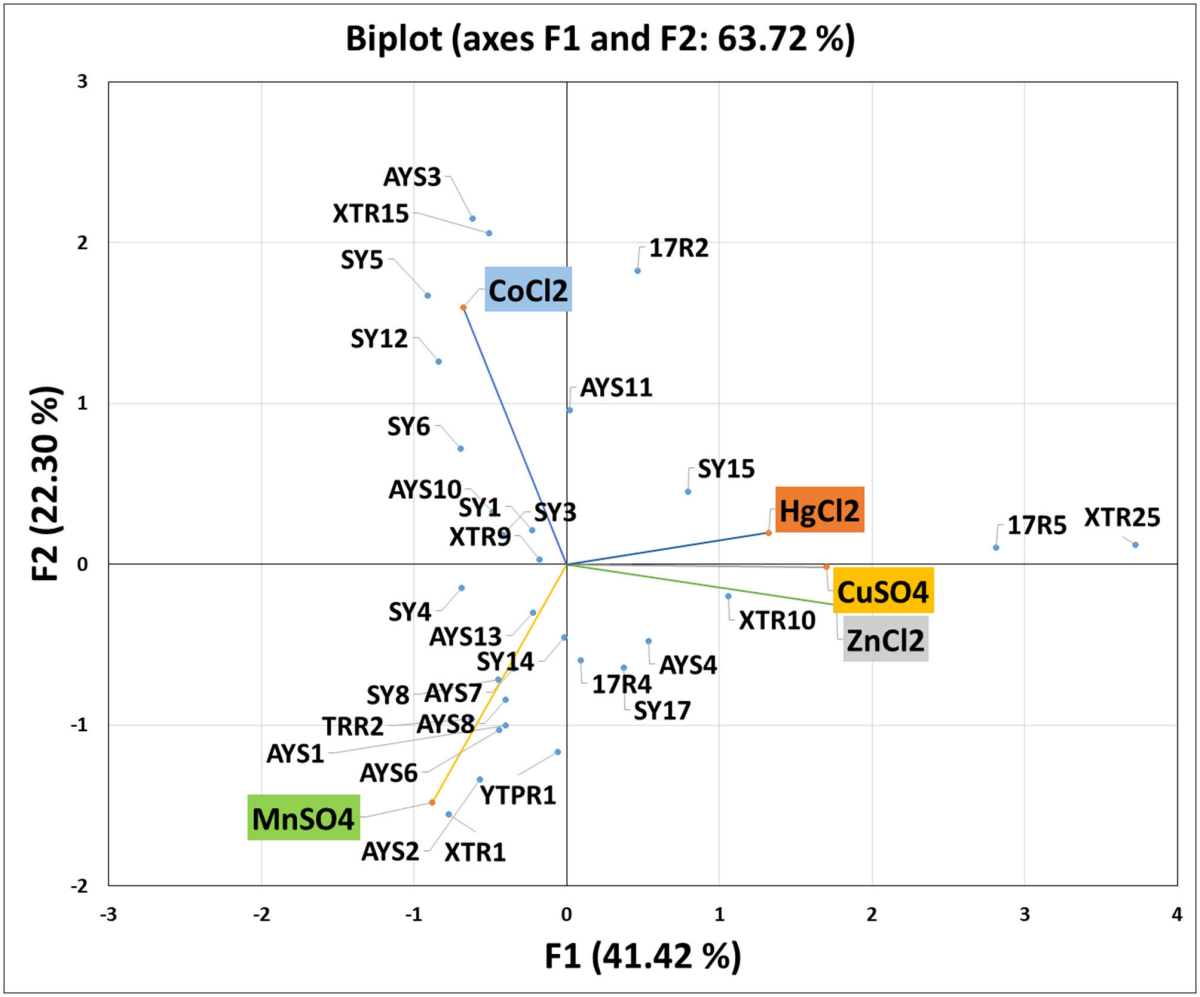
Principal component analysis showing the correlation of heavy metals and isolated strains.

Heavy metal PCA was calculated, and the relationship between these heavy metals and isolated strains was investigated. Table 4 shows that the first two Eigenvalues among the five main components had >1 values of 2.071 and 1.115, respectively. The five main components have a total variance of 100%. The test for the null hypothesis that the correlation matrix has an identity matrix was performed using Bartlett's sphericity. Taking this into account, these examinations provide the bare minimum for moving on to factor analysis. This test yielded a *p*-value of 0.0097 (a threshold value of 0.05), indicating that the factor analysis is valid and significant (Table 4). The results of PCA were comparable with those of MIC and EC50 analyses (Figure 4). The two principal components F1 and F1 demonstrate a substantial association among the heavy metals and isolates investigated, according to PCA. CuSO_4_, ZnCl_2_, and HgCl_2_ all had a favorable relationship. MnSO_4_ and CoCl_2_ were, however, only distantly connected. Individual heavy metals and highly resistant strains to their associated heavy metals were found to have a positive link when it came to isolated strains. In the area of the concerned heavy metal, the tolerant strains were present. For example, the presence of SY17, XTR10, and XTR25 in the proximity of Zn is positively correlated, indicating that these isolates are Zn tolerant. Mn tolerance is also seen in isolates ASY1, AYS2, TRR2, YTPR1, and XTR1. As demonstrated in Figure 4, the same is true for the other variables investigated. Based on MIC data in broth, it can be deduced that the isolates demonstrated the maximum tolerance to Cu > Zn > Mn > Co > Hg in our investigation.

**Table 4:** Estimated EC50 values for bacterial isolates.

**Estimated EC50 values for strains**					
**Strains**	**CuSO**_**4**_	**MnSO**_**4**_	**CoCl**_**2**_	**ZnCl**_**2**_	**HgCl**_**2**_
SY1	1.7	0.5334	0.117	0.2825	−0.8797
SY3	1.999	0.7855	0.107	−0.4687	−0.799
SY4	1.471	2.996	2.141	−0.2371	−0.9054
SY5	2.656	2.653	**11.79**	0.1253	−0.7718
SY6	2.681	2.791	**6.732**	0.1877	−0.6622
SY8	4.599	3.415	−0.1815	0.1149	−0.9025
SY12	**4.681**	3.187	**10.41**	0.08492	−0.5866
SY14	4.576	2.878	−0.2153	0.146	3.637
SY15	4.476	0.522	−0.08118	0.1338	**10.73**
SY17	1.997	2.564	0.00216	**3.594**	−0.9898
AYS1	**4.989**	**4.251**	−0.2131	0.5598	−0.8004
AYS2	4.146	**5.36**	−0.1864	0.4729	−0.9201
AYS3	2.299	0.7118	**11.21**	0.388	−0.3906
AYS4	**4.479**	3.345	−0.1997	0.5097	**9.823**
AYS6	4.401	**4.336**	−0.1939	0.5528	−0.9132
AYS7	2.266	3.335	0.07729	0.5512	−0.01096
AYS8	4.047	3.844	−0.05464	0.5139	−0.6595
AYS10	4.404	2.921	**4.882**	0.5389	−0.565
AYS11	4.032	2.291	**6.553**	0.5586	**5.679**
AYS13	**4.59**	2.068	−0.07472	0.4642	−0.678
XTR1	3.005	**6.66**	0.4827	8.77E−05	0.2805
XTR9	**4.886**	1.181	7.100e−001	6.80E−05	0.06013
17R2	0.1368	−4.453	0.000107	7.08E−05	**2.97**
TRR2	0.4644	**4.72**	0.4168	6.69E−05	0.7259
XTR15	2.03	3.06E−06	**9.245**	6.75E−05	0.2938
XTR10	**5.091**	1.018	0.1862	**5.01**	0.2903
XTR25	**38.52**	0.7225	0.5996	**7.078**	**13.63**
YTPR1	1.375	**4.727**	0.001713	2.107	0.951
17R4	**10.2**	2.989	8.26E−05	1.134	0.1367
17R5	**63.93**	7.36E−05	0.05073	**4.185**	0.1729

*Bold values indicate the maximum EC50 value obtained for a strain with respect to the heavy metal concentration*.

### ARG and MRG Analysis of the Genus *Geobacillus*

The investigation of ARGs revealed that the *Geobacillus* genus has no ARGs. The ARGs present in the *Geobacillus* genus were checked using four platforms, namely, CARD, Galaxy (with two applications: Galaxy ARGA and Galaxy ABRicate), and ARG-ANNOT NT. However, none of the platforms used yielded any results. This indicates that the *Geobacillus* genus lacks ARGs. As indicated in Supplementary Excel File 1, the MRG analysis revealed that all *Geobacillus* strains have MRGs. Against several MRGs, all of the strains had significant hits. This suggests that the genus *Geobacillus* is metal-resistant.

## Discussion

Over the course of the antibiotic era, environmental microorganisms that do not cause disease or are closely tied to antibiotic synthesis have been overlooked. They do, however, play a significant role in the evolution of antibiotic resistance. Antibiotic resistance in thermophiles or environmental bacteria is a significant issue. To address this, we used culture-dependent and culture-independent methodologies to examine bacterial diversity and antibiotic resistance profiles in four Sikkim hot springs. In terms of bacterial diversity, it was discovered that, aside from being geographically distinct, all hot springs have similar bacterial diversity, with Firmicutes as the dominant phylum. Many *Geobacillus* species were detected among the Firmicutes, but only a few *Bacillus* and *Anoxybacillus* species. Our prior research has also confirmed the Firmicutes' dominance in this ecosystem (Najar et al., 2018). Firmicutes predominate in Indian hot springs, which have temperatures ranging from 50 to 70°C. In addition, the phylum Firmicutes was shown to be the most adaptive in hot springs, which might be able to populate throughout a wide variety of temperature gradients (Zentgraf, 1992; Khalil, 2011; Deep et al., 2013; Panda and Sahu, 2013; Sen and Maiti, 2014; Daupan and Rivera, 2015; Baltaci et al., 2017; Mittal et al., 2017; Mohammad et al., 2017). It is possible that their dominance is attributable to the development of spores in Gram-positive bacteria (Boetius et al., 2015). Despite the fact that the majority of isolates were recognized as *Geobacillus* sp., the percentage similarity of several of these isolated bacteria was found to be over 95%, implying that these isolated bacteria are unique.

In a prior study, we used metagenomic methods to analyze the diversity of these hot springs (Najar et al., 2018, 2020b). Surprisingly, the metagenomic analysis revealed a majority of Proteobacteria, Actinobacteria, Bacteroidetes, and a small presence of Firmicutes (*Geobacillus* sp.). Despite their disparate geographic locations, the bacterial communities of these hot springs show less change. Yumthang and Reshi hot springs (Najar et al., 2020a,b) had a phylum-wise variety that was comparable with the other two Sikkim hot springs, Polok and Borong hot springs (Najar et al., 2018). Other hot springs in India (Badhai et al., 2015; Ghelani et al., 2015; Mangrola et al., 2015; Sangwan et al., 2015; Mehetre et al., 2016; Panda et al., 2016; Saxena et al., 2017) and China (Hedlund et al., 2012) had similar bacterial diversity, with abundant phyla, such as Proteobacteria. However, the percentages of different phyla differed among hot springs, which could be attributed to their geographic location.

Before the antibiotic era, the only habitats that were really immune to the effects of human antibiotic use were those that existed before the antibiotic period (Allen et al., 2010). The period before the introduction of sulphonamides, which happened in the late 1930s, is known as the “antibiotic ignorant” era because no antibiotics were produced industrially. In contrast, heavy metals have been used for illness treatment for millennia before antibiotics, and this may have favored genes encoding both heavy metal and antibiotic resistance (Baker-Austin et al., 2006). Resistance genes were found in bacteria that did not generate antibiotics before the medications became widely used, according to retrospective investigations (Dcosta et al., 2011). As a result, determinants of antibiotic resistance existed naturally and were likely prone to horizontal transfer even before the antibiotic era's severe selection pressure (Dcosta et al., 2011). This proclivity for resistance element genetic exchange is almost certain to have aided the rapid spread of antibiotic resistance in dangerous bacteria. Because there are few studies on antibiotic resistance among thermophilic bacteria, particularly those found in hot springs, this study was crucial to evaluate.

According to the results of the AST and MIC, all of the isolates collected from Sikkim's four hot springs are susceptible to at least 10 different antibiotics. The lack of PCR amplification of a few widely found ARGs, such as PBP1, PBP2, ampC, mecA1, and mecA2, using specific primers further suggests that these bacteria are vulnerable. The culturable bacteria's susceptibility could be related to a variety of factors, including fewer anthropogenic activities or the isolated character of hot springs, high temperatures deep within hot spring reservoirs, and reduced diversity and competition among microbial communities. Besides having more or less anthropogenic activities within the hot springs of Sikkim, thus the present study may suggest the school of thought that antibiotic resistance is mainly due to the environment. Thus, there is an evolutionary relationship between environment and accumulation of antibiotic resistance. In that case, anthropogenic activities may play a very less role in the emergence of antibiotic resistance in thermophiles. Firmicutes and Actinobacteria were shown to be minor hosts of ARGs and MRGs, and their abundance in the microbial community grew as the local environment's temperature rose (MacFadden et al., 2017). In contrast to other *Bacillaceae* members, *Geobacillus* sp. is one of the few bacteria that lack a large number of antibiotic marker genes. In the higher temperature range from 45 to 80°C at the surface level among the hot springs of Sikkim, the antibiotic susceptible nature of the thermophilic isolates was found. This may be also influenced by higher temperatures beneath the earth in the plumbing systems of hot springs, as no antibiotic resistance was found in any of the four hot springs with varying temperatures. Thus, it was less difficult to correlate the influence of temperature on antibiotic resistance. However, in a broader term, it may be suggested that an increase in temperature decreases antibiotic resistance. This susceptible nature of thermophiles was shown by our earlier metagenomic study which suggested that there was no ARGs representative of thermophiles (Najar et al., 2020a). Thus, these studies favor the argument that antibiotic resistance decreases with the elevation in temperatures.

In this investigation, we looked for ARGs in the type species of the *Geobacillus* genus and five of our randomly chosen *Geobacillus* isolates. All of the type species of *Geobacillus*, including our isolates, were verified to be ARG-free using four important and reliable online pipelines. Furthermore, the lack of any report on antibiotic-resistant *Geobacillus* sp. from a thermophilic environment, together with our findings, suggests that *Geobacillus* species are antibiotic susceptible natively or in general. Surprisingly, the RAST-based whole genome annotation results discovered a variety of basic resistance genes from a few classes, including -lactams and fluoroquinolones, in addition to the four processes utilized. However, these few genes were only presumptive because they did not match any known genes in BLAST and had no matches when tested in ARDA/CARD BLAST. As a result, these genes with alternative functions can be thought of as hypothetical and inactive in terms of antibiotic resistance. In addition, because hot springs are unique ecosystems with fewer anthropogenic activities, they may be absent from stable antibiotics at high temperatures. As a result of the lack of antibiotics, these thermophilic microorganisms may be under less pressure or competition to acquire ARGs. This may also be confirmed by the fact that thermophilic bacteria have smaller genomes than mesophilic bacteria. Based on our earlier investigation, metagenomic analysis of ARGs in the hot springs of Sikkim revealed a limited presence of ARGs, with most of them showing maximal identity, i.e., >97%, with Gram-negative and mesophilic bacteria (Najar et al., 2020a). As a result, the genes discovered through metagenomics may be the result of contamination of the soil microflora surrounding the topsoil layers of the hot spring.

**Figure 5:**
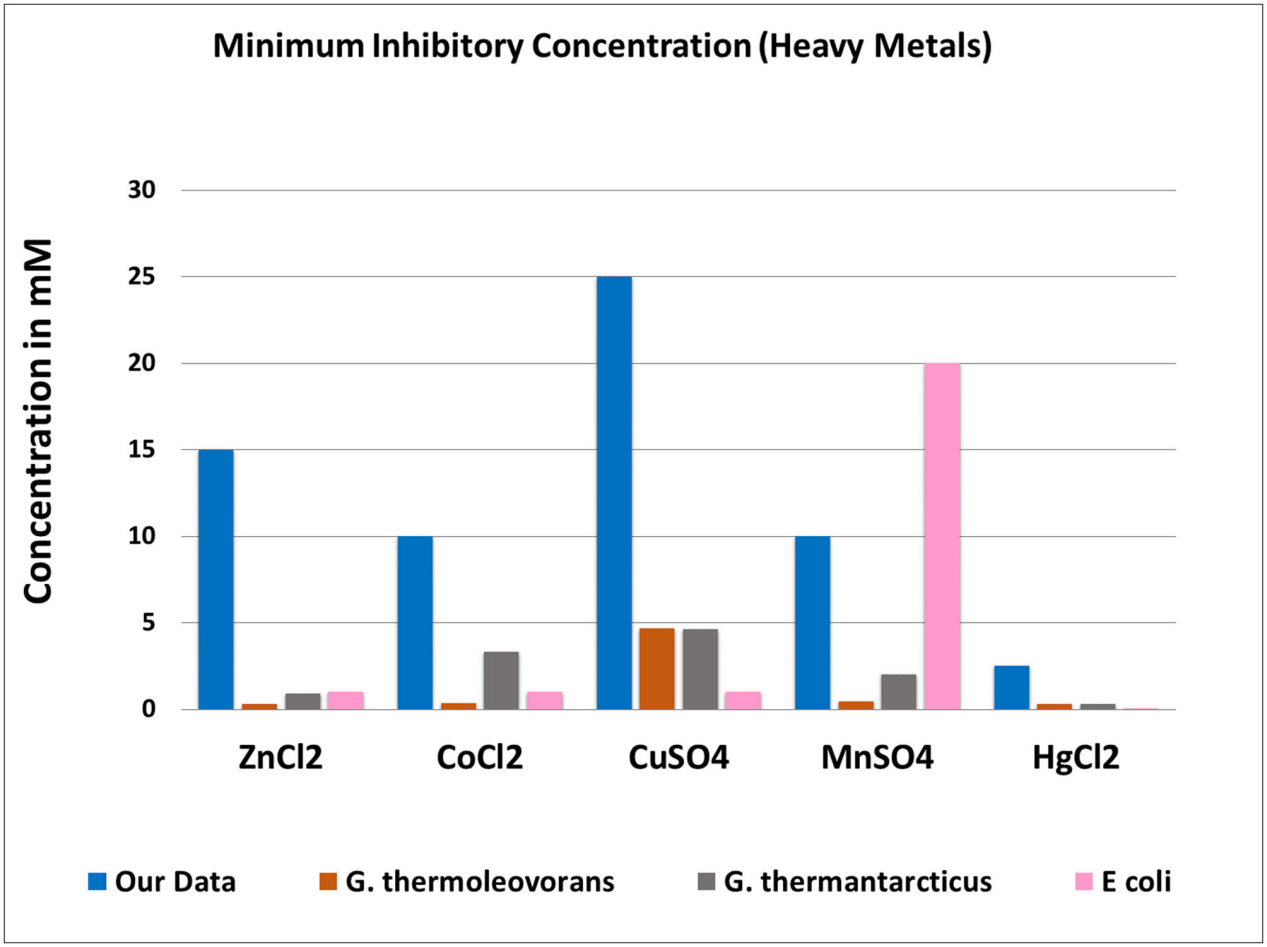
Comparison of minimum inhibitory concentrations among studied isolates and two known *Geobacillus s*pecies and an *E. coli*.

The contemplation of this uncertainty led us to look for HMRGs, as several researchers have found a close link between antibiotic resistance and heavy metal resistance. As a result, we first tested our bacterial isolates for metal tolerance (if any). Metal tolerance was also examined to see if any thermophilic isolates were tolerant to certain heavy metals and may be used in bioremediation and to see whether heavy metal resistance and antibiotic resistance co-occurred. Many of the isolates were found to be heavy metal tolerant, with MICs greater than those of *Escherichia coli* (Nies, 2000). When compared to the other two *Geobacillus* species, *G*. *thermoleovorans* and *G*. *thermantarcticus* (Poli et al., 2009), our isolates showed significantly higher metal resistance (Figure 5).

The metal resistance in all *Geobacillus* species and our randomly selected five *Geobacillus* isolates is further supported by whole genome sequencing results. Using the BecMet pipeline, multiple MRGs were discovered in almost all of the *Geobacillus* isolates tested, as shown in Supplementary Excel File 1. Similarly, HMRGs were discovered in our prior study's functional metagenomic investigation (Najar et al., 2020b). The hot spring water emerges from deep underground reservoirs through fissures surrounded by diverse types of rocks and metal pollutants; therefore, the culturable bacteria's metal tolerance may be related to varying element concentrations in the hot springs. Cu and Zn are plentiful in ecology, and the sources of these heavy metals identified in environmental samples are numerous. These heavy metals have been directly linked to the development of heavy metal tolerance in environmental microflora since the dawn of time (Wales and Davies, 2015; Poole, 2017). Cu and Zn have always been observed in environmental samples, together with the occurrence of antibiotic-resistant microbial populations (Knapp et al., 2011; Becerra-Castro et al., 2015). Cu-dependent ARGs' potential to transfer mobile genetic elements (MGEs) increases as Cu concentration rises, implying that Cu-dependent ARGs will be more easily accessible or mobile (Hu et al., 2016). There have been some fascinating results showing that the presence of Cu at low levels, such as sub-toxic metal, has a favorable effect on specific ARGs (Knapp et al., 2017).

Antibiotics can form strong Metallo-ligand coordination complexes with metal ions, and their ionic interaction can have a wide range of effects, such as duct ion in antibiotic efficacy (Weinberg, 1957; Poole, 2017). Eisner was the first to describe the Zn inactivation of penicillin by boosting lactam hydrolysis (Eisner and Porsecanski, 1946). Zn can bind to aminoglycosides, tetracycline, macrolides, vancomycin, quinolones, and a family of b-lactams and their derivatives (Poole, 2017). Vancomycin binds to Zn and induces the expression of Zn inducible genes in bacteria such as *E*. *coli, Bacillus subtilis*, and *Streptomyces* sp., implying that they need this trace metal (Zarkan et al., 2016). Cu has also been demonstrated to bind to penicillin and induce hydrolysis of this -lactam (Eisner and Porsecanski, 1946; Cressman et al., 1966). Cu binds to aminoglycosides, tetracyclines, chloramphenicol, novobiocin, macrolides, isoniazid, quinolones, vancomycin, and a range of -lactams, including cephalosporins, penicillins, and streptomycin and neomycin activity (Poole, 2017).

Because the MRGs were detected in culturable thermophilic bacteria and complete genomes of *Geobacillus* type species in this work, rather than their antibiotic counterparts, it is possible that there is no co-occurrence or co-selection of these genes in such isolated settings. However, this is a broad assumption that should be tested in other similar ecosystems. Similarly, the absence of antibiotic-resistant bacteria in four Sikkim hot springs and ARGs from thermophilic bacteria detected by either culture-dependent or culture-independent techniques suggest that thermophilic environments, such as hot springs and/or thermophilic Firmicutes (*Geobacillus*), are generally devoid of ARGs, supporting the hypothesis that anthropogenic activities were responsible for the emergence of antibiotic-resistant bacteria.

## Conclusion

Antibiotic resistance possesses a threat to humanity, yet the discovery of novel parvomes or the development of new antibiotic classes has been critical in the current antibiotic era. The origins and evolution of resistomes are unknown, necessitating a greater focus on researching the ARGs pattern for future lateral genetic transfer or horizontal gene transfer within the microbial population, which could cause global havoc. Metagenomics and whole genome sequencing analysis are vital in interpreting uncultivable microbial diversity and hunting for ARGs and MRGs in varied environments using functional methods through gene ontology (GO). The existence of MRGs among culturable and non-culturable bacteria from Sikkim's hot springs suggests the absence of co-occurrence or co-selection of ARGs and MRGs in these settings, in contrast to their antibiotic counterparts. Similarly, the presence of antibiotic susceptible bacteria in four Sikkim hot springs and the absence of ARGs from thermophilic bacteria detected by either culture-dependent or culture-independent techniques suggest that thermophilic environments, such as hot springs and/or thermophilic Firmicutes, primarily genus *Geobacillus*, are generally devoid of ARGs, supporting the hypothesis that anthropogenic activities were responsible for emergent antibiotic resistance. The hypothesis of associating temperature with the retention of ARGs cannot be concluded in this investigation because the bacteria found in these hot springs are not antibiotic-resistant. However, because antibiotic-susceptible bacteria were found in abundance in these hot springs, there may be less competition among these bacteria in these settings, resulting in a lower likelihood of ARG retention in these thermophilic bacteria. ARGs can undoubtedly be traced back to pristine landscapes and their micro-flora as an antediluvian notion; however, abiotic factors, such as anthropogenic activity, wind dispersal, and feces, can also be cofactors in the distribution of resistomes. This type of research in less antibiotic-exposed settings, such as thermal vents and high-temperature oil fields, could offer further light on this area.

## Data Availability Statement

The datasets presented in this study can be found in online repositories. The names of the repository/repositories and accession number(s) can be found below: https://www.ncbi.nlm.nih.gov/, NWUZ00000000 https://www.ncbi.nlm.nih.gov/, VRMH00000000 https://www.ncbi.nlm.nih.gov/, VRMI00000000 https://www.ncbi.nlm.nih.gov/, QCWL00000000.

## Author Contributions

INN did the experimental works, analysis, and prepared the manuscript. SD, SK, PS, KM, and MS helped in the sample collection, field study, and preparation of manuscript. NT designed the study, reviewed, and edited the manuscript. All authors contributed to the article and approved the submitted version.

## Funding

The work was funded by the Department of Biotechnology, Government of India (BT/PR25092/NER/95/1009/2017).

## Conflict of Interest

The authors declare that the research was conducted in the absence of any commercial or financial relationships that could be construed as a potential conflict of interest.

## Publisher's Note

All claims expressed in this article are solely those of the authors and do not necessarily represent those of their affiliated organizations, or those of the publisher, the editors and the reviewers. Any product that may be evaluated in this article, or claim that may be made by its manufacturer, is not guaranteed or endorsed by the publisher.
