# Supplementary figures and images for: Coexistence of Heavy Metal Tolerance and Antibiotic Resistance in Thermophilic Bacteria Belonging to Genus Geobacillus
Source: Front Microbiol. 2022 Aug 25;13:914037. doi: 10.3389/fmicb.2022.914037 (PMC9469766; doi:10.3389/fmicb.2022.914037)

**Supplementary Fig.1a, b, c. PCR amplifications of antibiotic resistance genes**

**
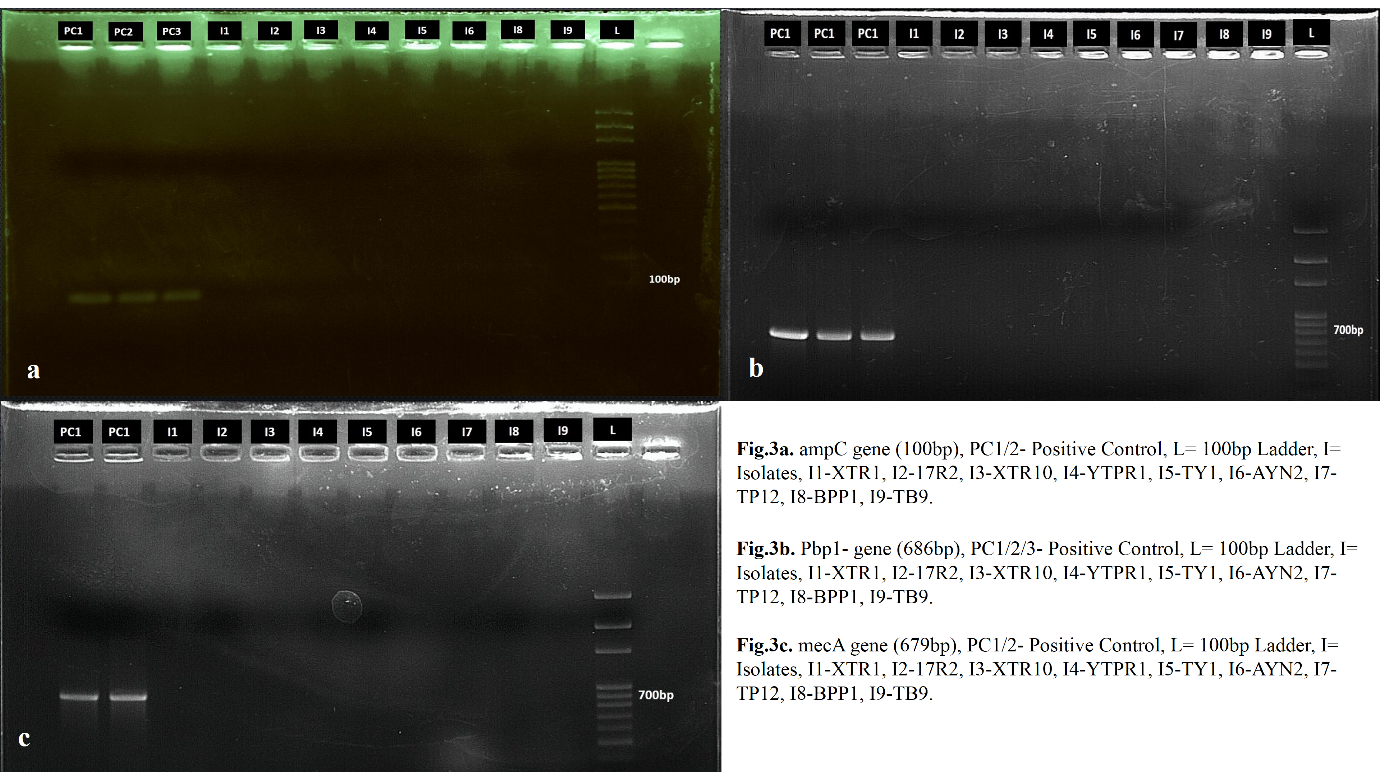
**

Supplement: Supplementary file 1 [file Data_Sheet_1.docx]
