# Supplementary material for: Coexistence of Heavy Metal Tolerance and Antibiotic Resistance in Thermophilic Bacteria Belonging to Genus Geobacillus
Source: Front Microbiol. 2022 Aug 25;13:914037. doi: 10.3389/fmicb.2022.914037 (PMC9469766; doi:10.3389/fmicb.2022.914037)
Supplement: Supplementary file 2 [file Data_Sheet_2.docx]

| **Thermus Agar (TA)** | g L^-1^ | **Modified Luria Bertani Agar** | g L^-1^ |
| --- | --- | --- | --- |
| Peptone | 8 | Tryptone | 5 |
| Yeast Extract | 4 | Yeast Extract | 5 |
| NaCl | 2 | NaCl | 3 |
| Agar | 25 | NaOH | 1 |
|  |  | MgSO4 | 1 |
| **Nutrient Agar** | g L^-1^ | CaCl2 | 1 |
| Peptone | 5 | FeSO4 | 1 |
| NaCl | 5 |  |  |
| Yeast Extract | 1.5 | **YTP-2 Medium** | g L^-1^ |
| Beef Extract | 1.5 | Tryptone | 2 |
| Agar | 25 | Yeast Extract | 2 |
|  |  | Sodium Pyruvate | 2 |
| **Luria Bertani Agar** | g L^-1^ | KCl | 1 |
| Casien Enzyme Hydrolysate | 10 | KNO_3_ | 2 |
| Yeast Extract | 5 | Na_2_HPO4 | 2 |
| NaCl | 5 | MgSO4 | 1 |
| Agar | 25 | CaCl2 | 0.03 |
|  |  | Classified Tomato Juice | 2ml |
| **TR Medium** | g L^-1^ | Agar | 25 |
| Tryptone | 4 |  |  |
| Yeast Extract | 2 | **BP Medium** | g L^-1^ |
| NaCl | 1 | Peptone | 4 |
| CaCl_2_ | 0.4mM | Beef Extract | 4 |
| MgCl_2_ | 0.4mM | K_2_HPO4 | 3 |
| Agar | 25 | KH_2_PO4 | 1 |
|  |  | Agar | 25 |
| **GYT Medium** | g L^-1^ |  |  |
| Glucose | 15 | **Actinomycete Isolation Agar** | g L^-1^ |
| Yeast Extract | 10 | Sodium Caseinate | 2 |
| Tryptone | 8 | L-Asparagine | 0.1 |
| CaCO_3_ | 5 | Sodium Propionate | 4 |
| NaCl | 2 | Dipotassium Phosphate | 0.5 |
| Agar | 25 | Magnesium Sulphate | 0.1 |
|  |  | Ferrous Sulphate | 0.001 |
|  |  | Agar | 25 |

**Supplementary Table 1.** Various Media used for isolation of Thermophilic Bacteria

| **R-2A Agar** | g L^-1^ | **Starch Agar** | g L^-1^ |
| --- | --- | --- | --- |
| Casein Acid Hydrolysate | 0.5 | Peptone | 5 |
| Yeast Extract | 0.5 | Yeast Extract | 1.5 |
| Proteose Peptone | 0.5 | Beef Extract | 1.5 |
| Dextrose | 0.5 | Starch Soluble | 2 |
| Starch Soluble | 0.5 | NaCl | 5 |
| Dipotassium Phosphate | 0.3 | Agar | 25 |
| Magnesium Sulphate | 0.024 |  |  |
| Sodium Pyruvate | 0.3 | **Gelatin Agar** | g L^-1^ |
| Agar | 25 | Gelatin | 30 |
|  |  | Casien Enzyme Hydrolysate | 10 |
| **Skim Milk Agar** | g L^-1^ | NaCl | 5 |
| Casien Enzyme Hydrolysate | 5 | Agar | 25 |
| Yeast Extract | 2.5 |  |  |
| Dextrose | 1 | **Mueller-Hinton Agar** | g L^-1^ |
| Skim milk powder | 28 | Meat, infusion from | 300 |
| Agar | 25 | Casein Acid Hydrolysate | 17.5 |
|  |  | Starch | 1.5 |
| **Plate Count Agar** | g L^-1^ | Agar | 25 |
| Casien Enzyme Hydrolysate | 5 |  |  |
| Yeast Extract | 2.5 |  |  |
| Dextrose | 1 | **Urea Agar** | g L^-1^ |
| Agar | 25 | Urea | 20 |
|  |  | Peptone | 1 |
| **Carbohydrate Fermentation Broth** | g L^-1^ | Dextrose | 1 |
| Sugar | 5 | Monopotassium phosphate | 2 |
| Peptone | 10 | Phenol Red | 0.01 |
| NaCl | 5 | NaCl | 5 |
| Phenol Red | 0.018 | Agar | 25 |

**Supplementary Table 2a: Morphological characterization of isolated bacteria from Yumthang hot spring.**

| Yumthang | Staining | | | Colony Morphology | | | | |
| --- | --- | --- | --- | --- | --- | --- | --- | --- |
| ISOLATES | Simple Staining | Gram Staining | Spore Staining | Color | Shape | Margins | Elevation | Density |
| TY1 | Small rods | + | - | white | circular | entire | flat | opaque |
| TY2 | Small rods | + | - | white creamy | oval | entire | flat | opaque |
| TY3 | Small rods | + | - | white | circular | entire | flat | opaque |
| TY4 | Small rods | + | - | off white | circular | entire | flat | opaque |
| TY5 | Small rods | + | + | white | circular | entire | flat | opaque |
| TY6 | Small rods | + | - | yellowish | circular | entire | flat | opaque |
| TY7 | Large rods | + | - | white | circular | entire | flat | opaque |
| TY8 | Small rods | + | + | white creamy | circular | entire | flat | opaque |
| TY9 | Medium rods | + | - | yellowish | circular | entire | flat | opaque |
| TY10 | Medium rods | + | - | off yellow | circular | entire | flat | opaque |
| TYNT4 | Medium rods | + | - | white creamy | round undulate | entire | flat | opaque |
| TYNT6 | Medium rods | + | + | off yellow | circular | entire | flat | opaque |
| TYNT 10 | Small rods | + | + | white creamy | circular | entire | flat | opaque |
| LYNT1 | Small rods | + | - | white creamy | circular | entire | flat | opaque |
| LYNT2 | Small rods | + | - | white creamy | circular | entire | flat | opaque |
| LYNT3 | Large rods | + | + | white | undulate | entire | flat | opaque |
| LYNT5 | Small rods | + | + | white creamy | circular | entire | flat | opaque |
| LYNT9 | Small rods | + | + | white creamy | circular | entire | flat | opaque |
| LYNT10 | Medium rods | + | + | white creamy | circular | entire | flat | opaque |
| AYN2 | Medium rods | + | + | white | circular | entire | flat | opaque |
| CTRL1 | Small rods | + | - | off yellow | circular | entire | flat | opaque |
| CTRL2 | Small rods | + | - | off yellow | circular | entire | flat | opaque |
| CTRL3 | Small rods | + | - | white creamy | circular | entire | flat | opaque |
| CTRL4 | Medium Rod | + | + | white creamy | circular | entire | flat | opaque |
| CTRL5 | Medium rods | + | + | white creamy | circular | entire | flat | opaque |
| CTRL6 | Medium rods | + | + | off yellow | circular | entire | flat | opaque |
| CTRL7 | Large rods | + | + | white creamy | circular | entire | flat | opaque |

The criteria for long, medium, and short size of bacteria was long (>5µm), medium (between 2-4 µm) and small (<2µm).

**Table.2b. Morphological characterization of isolated bacteria from Reshi hot spring.**

| **Reshi** | **Staining** | | | **Colony Morphology** | | | | |
| --- | --- | --- | --- | --- | --- | --- | --- | --- |
| **ISOLATAS** | **Simple Staining** | **Gram Stain** | **Spore stain** | **Color** | **Shape** | **Margins** | **Elevation** | **Density** |
| XTR1 | medium rods | **+** | **-** | pale yellow | circular | entire | flat | opaque |
| XTR2 | medium rods | **+** | **-** | white | circular | entire | flat | opaque |
| XTR3 | medium rods | **+** |  | white | circular | entire | flat | opaque |
| XTR4 | medium rods | **+** | **+** | white | circular | entire | flat | opaque |
| XTR5 | medium rods | **+** | **+** | pale yellow | circular | entire | flat | opaque |
| XTR6 | large rods | **+** | **+** | pale yellow | circular | entire | flat | opaque |
| XTR7 | small rods | **+** | **+** | pale yellow | circular | entire | flat | opaque |
| XTR8 | small rods | **+** | **+** | white creamy | circular | entire | flat | opaque |
| XTR9 | small rods | **+** | **+** | white creamy | circular | entire | flat | opaque |
| XTR10 | large rods | **+** | **+** | white | circular | entire | flat | opaque |
| XTR11 | medium rods | **+** | **+** | white creamy | circular | entire | flat | opaque |
| XTR12 | medium rods | **+** | **+** | pale yellow | circular | entire | flat | opaque |
| XTR13 | large rods | **+** | **-** | white | circular | entire | flat | opaque |
| XTR14 | medium rods | **+** | **-** | white creamy | circular | entire | flat | opaque |
| XTR15 | large rods | **+** | **-** | white | circular | entire | flat | opaque |
| XTR16 | medium rods | **+** | **-** | white creamy | circular | entire | flat | opaque |
| XTR17 | large rods | **+** | **-** | pale yellow | circular | entire | flat | opaque |
| XTR18 | medium rods | **+** | **-** | white | circular | entire | flat | opaque |
| XTR19 | large rods | **+** | **-** | white creamy | circular | entire | flat | opaque |
| XTR20 | medium rods | **+** | **-** | pale yellow | circular | entire | flat | opaque |
| XTR21 | large rods | **+** | **+** | white creamy | circular | entire | flat | opaque |
| XTR22 | medium rods | **+** | **+** | white | circular | entire | flat | opaque |
| XTR23 | medium rods | **+** | **-** | white creamy | circular | entire | flat | opaque |
| XTR24 | small rods | **+** | **-** | pale yellow | circular | entire | flat | opaque |
| XTR25 | medium rods | **+** | **-** | pale yellow | circular | entire | flat | opaque |
| XTR26 | medium rods | **+** | **+** | white creamy | circular | entire | flat | Opaque |
| XTR27 | small rods | **+** | **+** | white | circular | undulate | flat | opaque |
| XTR28 | small rods | **+** | **-** | white | circular | entire | flat | opaque |
| XTR31 | medium rods | **+** | **-** | pale yellow | circular | entire | flat | opaque |
| XTR32 | medium rods | **+** | **-** | white | circular | entire | flat | opaque |
| XTR34 | medium rods | **+** | **+** | white | circular | entire | flat | opaque |
| XTR36 | medium rods | **+** | **+** | pale yellow | circular | entire | flat | opaque |
| XTR37 | small rods | **+** | **+** | pale yellow | circular | entire | flat | opaque |
| XTR38 | medium rods | **+** | **+** | pale yellow | circular | entire | flat | opaque |
| XTR39 | medium rods | **+** | **+** | white | circular | entire | flat | opaque |
| XTR40 | small rods | **+** | **-** | white creamy | circular | entire | flat | opaque |
| XTR41 | medium rods | **+** | **-** | pale yellow | circular | entire | flat | opaque |
| XTR51 | medium rods | **+** | **-** | pale yellow | circular | entire | flat | opaque |
| XTR52 | medium rods | **+** | **+** | pale yellow | circular | entire | flat | opaque |

The criteria for long, medium, and short size of bacteria was long (>5µm), medium (between 2-4 µm) and small (<2µm).

**Table.2c. Morphological characterization of isolated bacteria from Polok/Borong hot spring.**

| **Polok/**  **Borong** | **Staining** | | | **Growth on agar plates** | | | | |
| --- | --- | --- | --- | --- | --- | --- | --- | --- |
| **Isolates** | **Simple Stain** | **Gram Stain** | **Spore Stain** | **Colony colour** | **Shape** | **Margin** | **Elevation** | **Density** |
| **TP1** | Medium rods | + | + | White | circular | Entire | Flat | Opaque |
| **TP2** | Medium rods | + | + | White Creamy | circular | Entire | Flat | Opaque |
| **TP3** | Medium rods | + | + | Off White | circular | Entire | Flat | Translucent |
| **TP4** | Very Small rods | + | - | Off White | circular | Entire | Flat | Opaque |
| **TP5** | Small rods | + | + | Off White | circular | Entire | Flat | Opaque |
| **TP6** | Large rods | + | - | White Creamy | Circular | Entire | Flat | Opaque |
| **TP7** | Small rods | + | - | Pale Yellow | Circular | Entire | Flat | Opaque |
| **TP8** | Small rods | + | - | Off White | Circular | Entire | Flat | Opaque |
| **TP9** | Medium rods | + | - | Off White | Circular | Entire | Flat | Opaque |
| **TP10** | Medium rods | + | + | White | Circular | Entire | Flat | Opaque |
| **TP11** | Small rods | + | + | White | Circular | Entire | Flat | Opaque |
| **TP12** | Small rods | + | - | White Creamy | Circular | Entire | Flat | Opaque |
| **TP13** | Medium rods | + | - | White | Circular | Entire | Flat | Opaque |
| **BPP1** | Small rods | + | + | Off White | Circular | Entire | Flat | Opaque |
| **BPP2** | Medium rods | + | + | Off White | Circular | Entire | Flat | Opaque |
| **BPP3** | Large rods | + | - | Off White | Circular | Entire | Flat | Opaque |
| **BPP4** | Medium rods | + | - | Off White | Circular | Entire | Flat | Opaque |
| **BPP5** | Medium rods | + | - | Off White | Circular | Entire | Flat | Opaque |
| **10PHP1** | Large rods | + | + | White Creamy | Circular | Entire | Flat | Opaque |
| **10PHP2** | Small rods | + | - | White Creamy | Circular | Entire | Flat | Opaque |
| **10PHP3** | Small rods | + | + | White Creamy | Circular | Entire | Flat | Opaque |
| **10PHP4** | Medium rods | + | + | Off White | Circular | Entire | Flat | Opaque |
| **10PHP5** | Medium rods | + | - | Off White | Circular | Entire | Flat | Opaque |
| **TB1** | Medium rods | + | - | Off White | Circular | Entire | Flat | Opaque |
| **TB2** | Small rods | + | - | White | Circular | Entire | Flat | Opaque |
| **TB3** | Medium rods | + | + | White | Circular | Entire | Flat | Opaque |
| **TB4** | Medium rods | + | + | White | Circular | Entire | Flat | Opaque |
| **TB5** | Small rods | + | - | White | Circular | Entire | Flat | Opaque |
| **TB6** | Medium rods | + | + | Off White | Circular | Entire | Flat | Opaque |
| **TB7** | Medium rods | + | - | Off White | Circular | Entire | Flat | Opaque |
| **TB8** | Medium rods | + | - | Off White | Circular | Entire | Flat | Opaque |
| **TB9** | Medium rods | + | + | White | Circular | Entire | Flat | Opaque |
| **TB10** | Large rods | + | + | Off White | Circular | Entire | Flat | Opaque |
| **TB11** | Medium rods | + | + | White | Circular | Entire | Flat | Opaque |
| **TB12** | Small rods | + | - | Off White | Circular | Entire | Flat | Opaque |
| **BPB1** | Medium rods | + | + | Off White | Circular | Entire | Flat | Opaque |
| **BPB2** | Small rods | + | + | Off White | Circular | Entire | Flat | Opaque |
| **BPB3** | Small rods | + | + | Off White | Circular | Entire | Flat | Opaque |
| **BPB4** | Small rods | + | + | Off White | Circular | Entire | Flat | Opaque |
| **BPB5** | Medium rods | + | + | Off White | Circular | Entire | Flat | Opaque |
| **17B1** | Long rods | + | + | White | Circular | Entire | Flat | Opaque |
| **TRB1** | Small rods | + | + | Off White | Circular | Entire | Flat | Opaque |
| **TRB2** | Small rods | + | - | White Creamy | Circular | Entire | Flat | Opaque |
| **YTPB1** | Medium rods | + | - | White Creamy | Circular | Entire | Flat | Opaque |
| **YTPB2** | Medium rods | + | - | Off White | Circular | Entire | Flat | Opaque |

The criteria for long, medium, and short size of bacteria was long (>5µm), medium (between 2-4 µm) and small (<2µm).

**Supplementary Table.3a. Antibiotic Susceptibility of Yumthang and Reshi isolates.**

|  | Antibiotic Susceptibility (mm) | | | | | | | | |
| --- | --- | --- | --- | --- | --- | --- | --- | --- | --- |
| ISOLATES | **Erythromycin**  **15mcg** | **Methicillin 10mcg** | **Penicillin 10U** | **Clindamycin 2mcg** | **Gentamycin 10mcg** | **Chloramphenicol**  **30mcg** | **Norfloxacin 10mcg** | **Ciprofloxacin**  **10mcg** | **Amoxicillin 10mcg** |
| TY1 | 28 | 19 | 35 | 32 | 27 | 23 | 31 | 26 | 37 |
| TY2 | 23 | 20 | 31 | 30 | 23 | 23 | 27 | 22 | 39 |
| TY3 | 27 | 21 | 35 | 30 | 26 | 21 | 30 | 25 | 36 |
| TY4 | 32 | 20 | 37 | 33 | 26 | 26 | 31 | 25 | 34 |
| TY5 | 35 | 23 | 38 | 35 | 31 | 29 | 32 | 27 | 37 |
| TY6 | 30 | 23 | 37 | 33 | 26 | 26 | 28 | 29 | 38 |
| TY7 | 31 | 21 | 35 | 32 | 26 | 23 | 30 | 24 | 39 |
| TY8 | 30 | 28 | 36 | 32 | 27 | 27 | 27 | 23 | 39 |
| TY9 | 32 | 27 | 37 | 30 | 29 | 26 | 29 | 25 | 36 |
| TY10 | 34 | 25 | 35 | 30 | 26 | 25 | 36 | 27 | 38 |
| TYNT4 | 29 | 39 | 32 | 29 | 24 | 23 | 33 | 24 | 34 |
| TYNT6 | 33 | 23 | 32 | 30 | 23 | 24 | 23 | 21 | 36 |
| TYNT 10 | 25 | 28 | 29 | 29 | 26 | 24 | 32 | 25 | 34 |
| LYNT1 | 30 | 39 | 35 | 32 | 27 | 23 | 32 | 25 | 38 |
| LYNT2 | 26 | 40 | 34 | 32 | 27 | 24 | 27 | 24 | 38 |
| LYNT3 | 27 | 36 | 30 | 28 | 22 | 21 | 30 | 23 | 28 |
| LYNT5 | 31 | 37 | 33 | 29 | 25 | 23 | 32 | 28 | 32 |
| LYNT9 | 34 | 35 | 35 | 32 | 25 | 25 | 30 | 30 | 39 |
| LYNT10 | 28 | 35 | 35 | 28 | 27 | 24 | 24 | 29 | 34 |
| AYN2 | 26 | 18 | 31 | 31 | 25 | 22 | 22 | 23 | 28 |
| XTR1 | 29 | 34 | 33 | 29 | 26 | 26 | 33 | 25 | 38 |
| XTR2 | 33 | 38 | 32 | 34 | 23 | 24 | 29 | 33 | 40 |
| XTR3 | 34 | 36 | 34 | 27 | 24 | 22 | 31 | 28 | 37 |
| XTR4 | 32 | 38 | 34 | 30 | 27 | 25 | 35 | 33 | 40 |
| XTR5 | 27 | 39 | 29 | 29 | 20 | 28 | 31 | 32 | 34 |
| XTR6 | 32 | 32 | 33 | 30 | 25 | 30 | 33 | 25 | 38 |
| XTR7 | 32 | 37 | 34 | 27 | 22 | 26 | 30 | 31 | 36 |
| XTR8 | 29 | 36 | 32 | 27 | 23 | 25 | 33 | 27 | 37 |
| XTR9 | 31 | 33 | 31 | 26 | 26 | 28 | 33 | 34 | 36 |
| XTR10 | 30 | 34 | 40 | 26 | 24 | 28 | 38 | 32 | 31 |
| XTR11 | 31 | 36 | 32 | 26 | 22 | 23 | 32 | 32 | 39 |
| XTR12 | 33 | 31 | 34 | 29 | 21 | 23 | 36 | 27 | 40 |
| XTR13 | 30 | 34 | 30 | 27 | 22 | 27 | 32 | 33 | 40 |
| XTR14 | 32 | 38 | 34 | 27 | 25 | 24 | 30 | 27 | 38 |
| XTR15 | 31 | 32 | 26 | 29 | 21 | 26 | 27 | 25 | 34 |
| XTR16 | 33 | 33 | 32 | 31 | 24 | 27 | 36 | 33 | 40 |
| XTR17 | 32 | 37 | 32 | 31 | 25 | 28 | 36 | 35 | 39 |
| XTR18 | 36 | 40 | 30 | 32 | 26 | 26 | 38 | 33 | 30 |
| XTR19 | 32 | 34 | 32 | 28 | 24 | 29 | 31 | 31 | 38 |
| XTR20 | 32 | 36 | 32 | 30 | 26 | 22 | 32 | 29 | 37 |
| XTR21 | 31 | 37 | 33 | 32 | 25 | 30 | 34 | 34 | 40 |
| XTR22 | 29 | 32 | 39 | 31 | 28 | 34 | 37 | 38 | 36 |
| XTR23 | 25 | 33 | 29 | 30 | 26 | 30 | 37 | 38 | 40 |
| XTR24 | 33 | 31 | 37 | 28 | 22 | 29 | 34 | 33 | 40 |
| XTR25 | 29 | 34 | 31 | 32 | 32 | 25 | 35 | 37 | 40 |
| XTR26 | 28 | 38 | 25 | 27 | 23 | 28 | 32 | 35 | 38 |
| XTR27 | 29 | 32 | 28 | 27 | 23 | 24 | 35 | 33 | 39 |
| XTR28 | 28 | 33 | 27 | 27 | 22 | 29 | 31 | 32 | 38 |
| XTR29 | 28 | 37 | 27 | 28 | 23 | 28 | 31 | 32 | 37 |
| XTR30 | 28 | 40 | 29 | 29 | 26 | 25 | 35 | 35 | 40 |
| XTR31 | 28 | 34 | 27 | 30 | 23 | 26 | 34 | 36 | 40 |
| XTR32 | 30 | 36 | 29 | 29 | 23 | 27 | 38 | 37 | 40 |
| XTR33 | 35 | 37 | 29 | 31 | 23 | 27 | 34 | 34 | 40 |
| XTR34 | 31 | 32 | 30 | 34 | 23 | 32 | 33 | 31 | 40 |
| XTR35 | 29 | 33 | 32 | 32 | 23 | 28 | 39 | 38 | 40 |
| XTR36 | 29 | 35 | 28 | 28 | 27 | 32 | 35 | 35 | 40 |
| XTR37 | 35 | 31 | 35 | 32 | 29 | 31 | 36 | 36 | 40 |
| XTR38 | 31 | 29 | 29 | 28 | 22 | 25 | 32 | 38 | 39 |

**Table.3b. Antibiotic Susceptibility of Polok and Borong isolates.**

|  | Antibiotic Susceptibility | | | | | | | | | |
| --- | --- | --- | --- | --- | --- | --- | --- | --- | --- | --- |
| ISOLATES | **Erythromycin 15mcg** | **Methicillin 10mcg** | **Penicillin 10U** | **Clindamycin 2mcg** | **Gentamycin 10mcg** | **Chloramphenicol 30mcg** | **Norfloxacin 10mcg** | **Ciprofloxacin 10mcg** | **Ampicillin 10mcg** |  |
| TP1 | 27 | 27 | 28 | 29 | 27 | 34 | 22 | 32 | 36 |  |
| TP2 | 27 | 30 | 30 | 29 | 29 | 22 | 30 | 30 | 39 |  |
| TP3 | 25 | 25 | 26 | 31 | 29 | 28 | 39 | 34 | 26 |  |
| TP4 | 25 | 26 | 27 | 22 | 25 | 24 | 26 | 28 | 31 |  |
| TP5 | 26 | 28 | 26 | 25 | 24 | 26 | 27 | 28 | 36 |  |
| TP6 | 28 | 27 | 28 | 28 | 27 | 31 | 31 | 26 | 27 |  |
| TP7 | 26 | 26 | 24 | 26 | 28 | 27 | 27 | 28 | 25 |  |
| TP8 | 31 | 25 | 29 | 31 | 26 | 27 | 28 | 29 | 25 |  |
| TP9 | 27 | 30 | 28 | 30 | 24 | 27 | 29 | 31 | 33 |  |
| TP10 | 31 | 30 | 25 | 23 | 29 | 28 | 25 | 29 | 31 |  |
| TP11 | 26 | 27 | 27 | 29 | 24 | 26 | 31 | 32 | 36 |  |
| TP12 | 26 | 24 | 26 | 23 | 29 | 24 | 26 | 27 | 30 |  |
| TP13 | 24 | 28 | 28 | 27 | 26 | 27 | 29 | 28 | 40 |  |
| BPP1 | 27 | 26 | 26 | 30 | 26 | 25 | 28 | 27 | 33 |  |
| BPP2 | 23 | 24 | 37 | 28 | 18 | 26 | 27 | 30 | 34 |  |
| BPP3 | 32 | 31 | 32 | 30 | 35 | 27 | 35 | 29 | 35 |  |
| BPP4 | 37 | 28 | 18 | 28 | 27 | 30 | 26 | 29 | 24 |  |
| BPP5 | 25 | 29 | 24 | 25 | 28 | 36 | 24 | 36 | 28 |  |
| TB1 | 29 | 32 | 34 | 27 | 27 | 27 | 34 | 34 | 36 |  |
| TB2 | 38 | 34 | 30 | 27 | 28 | 27 | 28 | 28 | 36 |  |
| TB3 | 25 | 29 | 29 | 29 | 24 | 26 | 28 | 28 | 29 |  |
| TB4 | 25 | 30 | 25 | 29 | 24 | 27 | 35 | 29 | 35 |  |
| TB5 | 30 | 30 | 31 | 28 | 27 | 27 | 31 | 30 | 39 |  |
| TB6 | 22 | 27 | 27 | 27 | 22 | 23 | 25 | 29 | 34 |  |
| TB7 | 27 | 30 | 25 | 29 | 24 | 25 | 26 | 28 | 37 |  |
| TB8 | 27 | 27 | 28 | 27 | 20 | 25 | 28 | 27 | 30 |  |
| TB9 | 25 | 27 | 26 | 28 | 22 | 22 | 25 | 28 | 36 |  |
| TB10 | 27 | 29 | 28 | 31 | 24 | 27 | 25 | 28 | 34 |  |
| TB11 | 27 | 26 | 29 | 24 | 30 | 28 | 27 | 25 | 29 |  |
| TB12 | 22 | 24 | 36 | 28 | 25 | 27 | 27 | 24 | 26 |  |
| BPB1 | 28 | 29 | 25 | 28 | 24 | 24 | 24 | 29 | 31 |  |
| BPB2 | 28 | 30 | 26 | 29 | 24 | 24 | 25 | 27 | 33 |  |
| BPB3 | 27 | 25 | 24 | 36 | 28 | 31 | 26 | 37 | 35 |  |
| BPB4 | 24 | 26 | 28 | 28 | 23 | 25 | 27 | 26 | 35 |  |

**Table.3c. Antibiotic susceptibility pattern of *Geobacillus* *thermoleovorans* (MTCC4219) *and Geobacillus stearothermophilus* (MTCC37)**

| Antibiotic susceptibility of two known *Geobacillus* species | | |
| --- | --- | --- |
| Antibiotics | ***Geobacillus thermoleovorans*** | ***Geobacillus stearothermophilus*** |
| Erythromycin | 29 | 33 |
| Methicillin | 33 | 32 |
| Clindamycin | 35 | 30 |
| Ampicillin | >40 | 35 |
| Chloramphenicol | 29 | 26 |
| Penicillin | >40 | 40 |

**Table.4a.** Minimum inhibitory concentration of erythromycin.

|  | **MIC ERYTHROMYCIN (µg ml^-1^)** | | | | | |
| --- | --- | --- | --- | --- | --- | --- |
| **SNO.** | **0.25** | **0.5** | **1** | **2** | **4** | **8** |
| AYS8 | G | NG | NG | NG | NG | NG |
| AYS10 | G | G | G | NG | NG | NG |
| AYN2 | G | G | G | NG | NG | NG |
| SY1 | G | G | NG | NG | NG | NG |
| SY3 | G | G | G | NG | NG | NG |
| TY1 | G | G | G | NG | NG | NG |
| XTR9 | G | G | G | NG | NG | NG |
| XTR10 | G | G | G | NG | NG | NG |
| XTR4 | G | G | G | NG | NG | NG |
| XTR3 | G | G | G | NG | NG | NG |
| XTR1 | G | G | G | NG | NG | NG |
| XTR15 | G | G | G | NG | NG | NG |
| TP1 | G | G | G | NG | NG | NG |
| BPP1 | G | G | G | NG | NG | NG |
| BPP2 | G | G | NG | NG | NG | NG |
| TB2 | G | G | NG | NG | NG | NG |
| TB3 | G | G | G | NG | NG | NG |
| TB9 | G | G | G | NG | NG | NG |

G=growth, NG=No Growth.

**Table.4b.** Minimum inhibitory concentration of vancomycin.

|  | **MIC VANCOMYCIN (µg ml^-1^)** | | | | | |
| --- | --- | --- | --- | --- | --- | --- |
| **SNO.** | **0.25** | **0.5** | **1** | **2** | **4** | **8** |
| **AYS8** | G | G | G | NG | NG | NG |
| **AYS10** | G | G | NG | NG | NG | NG |
| **AYN2** | G | G | NG | NG | NG | NG |
| **SY1** | G | G | NG | NG | NG | NG |
| **SY3** | G | G | NG | NG | NG | NG |
| **TY1** | G | G | NG | NG | NG | NG |
| **XTR9** | G | G | NG | NG | NG | NG |
| **XTR10** | G | G | NG | NG | NG | NG |
| **XTR4** | G | G | G | NG | NG | NG |
| **XTR3** | G | G | NG | NG | NG | NG |
| **XTR1** | G | G | G | NG | NG | NG |
| **XTR15** | G | G | NG | NG | NG | NG |
| **TP1** | G | G | NG | NG | NG | NG |
| **BPP1** | G | G | NG | NG | NG | NG |
| **BPP2** | G | G | G | NG | NG | NG |
| **TB2** | G | G | G | NG | NG | NG |
| **TB3** | G | G | G | NG | NG | NG |
| **TB9** | G | G | NG | NG | NG | NG |

G=Growth, NG=No Growth

**Table.4c.** Minimum inhibitory concentration of chloramphenicol.

|  | **MIC CHLORAMPHENECOL (µg ml^-1^)** | | | | | |
| --- | --- | --- | --- | --- | --- | --- |
| **SNO.** | **0.25** | **0.5** | **1** | **2** | **4** | **8** |
| **AYS8** | G | G | G | G | G | NG |
| **AYS10** | G | G | G | G | G | NG |
| **AYN2** | G | G | G | G | G | NG |
| **SY1** | G | G | G | G | G | NG |
| **SY3** | G | G | G | G | G | NG |
| **TY1** | G | G | G | G | NG | NG |
| **XTR9** | G | G | G | G | G | NG |
| **XTR10** | G | G | G | G | G | NG |
| **XTR4** | G | G | G | G | G | NG |
| **XTR3** | G | G | G | G | G | NG |
| **XTR1** | G | G | G | G | NG | NG |
| **XTR15** | G | G | G | G | G | NG |
| **TP1** | G | G | G | G | G | NG |
| **BPP1** | G | G | G | G | G | NG |
| **BPP2** | G | G | G | G | NG | NG |
| **TB2** | G | G | G | G | NG | NG |
| **TB3** | G | G | G | G | NG | NG |
| **TB9** | G | G | G | G | G | NG |

G=Growth, NG=No Growth

**Table.4d.** Minimum inhibitory concentration of penicillin.

|  | **MIC PENICILLIN (µg ml^-1^)** | | | | | |
| --- | --- | --- | --- | --- | --- | --- |
| **SNO.** | **0.125** | **0.25** | **0.5** | **1** | **2** | **4** |
| **AYS8** | NG | NG | NG | NG | NG | NG |
| **AYS10** | NG | NG | NG | NG | NG | NG |
| **AYN2** | G | NG | NG | NG | NG | NG |
| **SY1** | G | NG | NG | NG | NG | NG |
| **SY3** | G | NG | NG | NG | NG | NG |
| **TY1** | G | NG | NG | NG | NG | NG |
| **XTR9** | G | NG | NG | NG | NG | NG |
| **XTR10** | G | NG | NG | NG | NG | NG |
| **XTR4** | G | NG | NG | NG | NG | NG |
| **XTR3** | NG | NG | NG | NG | NG | NG |
| **XTR1** | G | NG | NG | NG | NG | NG |
| **XTR15** | G | NG | NG | NG | NG | NG |
| **TP1** | G | NG | NG | NG | NG | NG |
| **BPP1** | NG | NG | NG | NG | NG | NG |
| **BPP2** | G | NG | NG | NG | NG | NG |
| **TB2** | G | NG | NG | NG | NG | NG |
| **TB3** | G | NG | NG | NG | NG | NG |
| **TB9** | G | NG | NG | NG | NG | NG |

G=Growth, NG=No Growth

**Table.5.** Estimated EC50 values for bacterial isolates.

| **Estimated EC50 values for strains** | | | | | |
| --- | --- | --- | --- | --- | --- |
| **Strains** | **CuSO_4_** | **MnSO_4_** | **CoCl_2_** | **ZnCl_2_** | **HgCl_2_** |
| **SY1** | 1.7 | 0.5334 | 0.117 | 0.2825 | -0.8797 |
| **SY3** | 1.999 | 0.7855 | 0.107 | -0.4687 | -0.799 |
| **SY4** | 1.471 | 2.996 | 2.141 | -0.2371 | -0.9054 |
| **SY5** | 2.656 | 2.653 | **11.79** | 0.1253 | -0.7718 |
| **SY6** | 2.681 | 2.791 | **6.732** | 0.1877 | -0.6622 |
| **SY8** | 4.599 | 3.415 | -0.1815 | 0.1149 | -0.9025 |
| **SY12** | **4.681** | 3.187 | **10.41** | 0.08492 | -0.5866 |
| **SY14** | 4.576 | 2.878 | -0.2153 | 0.146 | 3.637 |
| **SY15** | 4.476 | 0.522 | -0.08118 | 0.1338 | **10.73** |
| **SY17** | 1.997 | 2.564 | 0.00216 | **3.594** | -0.9898 |
| **AYS1** | **4.989** | **4.251** | -0.2131 | 0.5598 | -0.8004 |
| **AYS2** | 4.146 | **5.36** | -0.1864 | 0.4729 | -0.9201 |
| **AYS3** | 2.299 | 0.7118 | **11.21** | 0.388 | -0.3906 |
| **AYS4** | **4.479** | 3.345 | -0.1997 | 0.5097 | **9.823** |
| **AYS6** | 4.401 | **4.336** | -0.1939 | 0.5528 | -0.9132 |
| **AYS7** | 2.266 | 3.335 | 0.07729 | 0.5512 | -0.01096 |
| **AYS8** | 4.047 | 3.844 | -0.05464 | 0.5139 | -0.6595 |
| **AYS10** | 4.404 | 2.921 | **4.882** | 0.5389 | -0.565 |
| **AYS11** | 4.032 | 2.291 | **6.553** | 0.5586 | **5.679** |
| **AYS13** | **4.59** | 2.068 | -0.07472 | 0.4642 | -0.678 |
| **XTR1** | 3.005 | **6.66** | 0.4827 | 8.77E-05 | 0.2805 |
| **XTR9** | **4.886** | 1.181 | 7.100e-001 | 6.80E-05 | 0.06013 |
| **17R2** | 0.1368 | -4.453 | 0.000107 | 7.08E-05 | **2.97** |
| **TRR2** | 0.4644 | **4.72** | 0.4168 | 6.69E-05 | 0.7259 |
| **XTR15** | 2.03 | 3.06E-06 | **9.245** | 6.75E-05 | 0.2938 |
| **XTR10** | **5.091** | 1.018 | 0.1862 | **5.01** | 0.2903 |
| **XTR25** | **38.52** | 0.7225 | 0.5996 | **7.078** | **13.63** |
| **YTPR1** | 1.375 | **4.727** | 0.001713 | 2.107 | 0.951 |
| **17R4** | **10.2** | 2.989 | 8.26E-05 | 1.134 | 0.1367 |
| **17R5** | **63.93** | 7.36E-05 | 0.05073 | **4.185** | 0.1729 |
